# Patterns of progression and survival in patients with mismatch repair deficient/ microsatellite instability- high metastatic colorectal cancer treated with immunotherapy

**DOI:** 10.1093/oncolo/oyag235

**Published:** 2026-07-20

**Authors:** Michael LaPelusa, Preksha Shah, Deepak Bhamidipati, Naishu Kui, Alisha Bent, Arvind Dasari, Ryan Huey, Bryan Kee, Scott Kopetz, Phat Le, Kaysia Ludford, Maria Pia Morelli, Van K Morris, Christine Parseghian, Kanwal Raghav, Jason Willis, Robert Wolff, Dan Zhao, Michael J Overman, Victoria S Higbie

**Affiliations:** Division of Cancer Medicine, MD Anderson Cancer Center, Houston, TX 77030, United States; Department of Gastrointestinal Medical Oncology, MD Anderson Cancer Center, Houston, TX 77030, United States; Sarah Cannon Research Institute, Nashville, TN 37203, United States; Department of Biostatistics, MD Anderson Cancer Center, Houston, TX 77030, United States; Department of Gastrointestinal Medical Oncology, MD Anderson Cancer Center, Houston, TX 77030, United States; Department of Gastrointestinal Medical Oncology, MD Anderson Cancer Center, Houston, TX 77030, United States; Department of Gastrointestinal Medical Oncology, MD Anderson Cancer Center, Houston, TX 77030, United States; Department of Gastrointestinal Medical Oncology, MD Anderson Cancer Center, Houston, TX 77030, United States; Department of Gastrointestinal Medical Oncology, MD Anderson Cancer Center, Houston, TX 77030, United States; Department of Gastrointestinal Medical Oncology, MD Anderson Cancer Center, Houston, TX 77030, United States; Department of Gastrointestinal Medical Oncology, MD Anderson Cancer Center, Houston, TX 77030, United States; Department of Gastrointestinal Medical Oncology, MD Anderson Cancer Center, Houston, TX 77030, United States; Department of Gastrointestinal Medical Oncology, MD Anderson Cancer Center, Houston, TX 77030, United States; Department of Gastrointestinal Medical Oncology, MD Anderson Cancer Center, Houston, TX 77030, United States; Department of Gastrointestinal Medical Oncology, MD Anderson Cancer Center, Houston, TX 77030, United States; Department of Gastrointestinal Medical Oncology, MD Anderson Cancer Center, Houston, TX 77030, United States; Department of Gastrointestinal Medical Oncology, MD Anderson Cancer Center, Houston, TX 77030, United States; Department of Gastrointestinal Medical Oncology, MD Anderson Cancer Center, Houston, TX 77030, United States; Department of Gastrointestinal Medical Oncology, MD Anderson Cancer Center, Houston, TX 77030, United States; Department of Gastrointestinal Medical Oncology, MD Anderson Cancer Center, Houston, TX 77030, United States

**Keywords:** colorectal cancer, MSI-H/dMMR, immunotherapy, immune checkpoint blockade

## Abstract

**Background:**

Immunotherapy has shown to be efficacious in patients with deficient mismatch repair (dMMR)/microsatellite instability-high (MSI-H) colorectal cancer (CRC). Unfortunately, there is still a population of patients who either do not respond or progress after prior response. Understanding the progression dynamics and outcomes based on the nature of disease progression (PD) is critical in this patient population.

**Methods:**

We performed a retrospective analysis of 166 patients with advanced dMMR/MSI-H CRC who received immunotherapy. PD patterns were classified as intrinsic (progression at first restaging scan) or adaptive (progression after initial stable/responding disease) and as single organ or systemic.

**Results:**

Progression was seen in 64 patients (38.6%) with single organ progression in 31 (48.4%) and systemic progression in 33 (51.5%). Intrinsic progression was seen in 32 patients (50%) and adaptive progression in 32 (50%). Patients with single organ progression had a longer median TTP (mTTP) and median OS (mOS) compared to patients with systemic progression (mTTP: 8.8 vs 4.0 months, *P* = .005; mOS: 65.9 vs 18.7 months, *P* = .023). Patients with adaptive PD had a longer mTTP and mOS compared to patients with intrinsic PD (mTTP: 11.6 vs 1.9 months, *P* = <.01; mOS: 65.9 months vs 17.0 months, *P* = .001).

**Conclusion:**

This analysis identifies significant differences in outcomes of patients with dMMR/MSI-H CRC based on the pattern and extent of progression, underscoring the need for tailored therapeutic strategies depending on the nature of PD-1-based progression.

Implications for Practice Although immune checkpoint inhibitors have shown impressive and often durable responses in dMMR/MSI-H colorectal cancer patients, there is still a substantial population of patients who progress on immunotherapy. Understanding of why some patients do not respond is limited. This study analyzes the differences in outcomes in patients based on the pattern and extent of progression, highlighting the important need for more tailored therapeutic strategies in these patients.

## Introduction

There are an estimated 150 000 new diagnoses of colorectal cancer (CRC) and over 53 000 CRC-related deaths in the United States annually.^1^ Approximately 15% of patients with CRC have deficient mismatch repair (dMMR) tumors—a distinct genetic subset of CRC. dMMR CRC can be sporadic, often stemming from somatic hyper-methylation of the *MLH1* gene promoter in the context of *BRAF V600E* alterations, or hereditary, which most frequently stems from pathogenic germline mutations in the mismatch repair pathway genes.^2–4^ Subsequently, cells lose the ability to identify and repair nucleotide substitution and insertion-deletion loops at microsatellite loci during DNA replication. As a result of altered microsatellite sequence lengths, these cells exhibit a phenotype known as microsatellite instability (MSI-H).^3^

Pembrolizumab, an antibody against programmed death receptor 1 (PD-1), monotherapy was shown to have an objective response rate (ORR) of 40% and a disease control rate (DCR) of 90% in KEYNOTE 016, a phase II trial in patients with pretreated advanced dMMR/MSI-H CRC.^5^^,^^6^ This was confirmed in KEYNOTE 164, a phase II trial in which an ORR of 33% was observed in 124 patients with pretreated, advanced dMMR/MSI-H CRC.^7^ In the frontline Phase III Keynote 177, patients with untreated advanced dMMR/MSI-H CRC who received pembrolizumab experienced longer median progression-free survival than patients who received chemotherapy (16.5 vs 8.2 months).^8^ Other immunotherapy agents have shown similar results in patients with advanced dMMR/MSI-H CRC such as Nivolumab, another anti-PD1, with or without ipilimumab, and anti-CTLA4, in the Checkmate 142 trial.^9^^,^^10^ In Checkmate 8HW, a phase 3 trial of nivolumab with ipilimumab or alone versus chemotherapy, Nivolumab with ipilimumab had a PFS at 2 years of 72% compared to 14% in the chemotherapy arm.^11^ Immunotherapy has also shown activity in patients with locally advanced dMMR/MSI-H rectal cancer. In a phase II study that evaluated dostarlimab monotherapy in patients with locally advanced dMMR/MSI-H rectal cancer, all 49 patients had a complete clinical response, with no evidence of tumor on magnetic resonance imaging, PET, endoscopic evaluation, digital rectal examination, or biopsy with 37 of these (76%) having sustained complete clinical response at 12 months.^12^ Another showed a major pathologic response (less than 10% viable tumor) was observed in 19 of 20 patients with early-stage colon cancer who received a single dose of ipilimumab and 2 doses of nivolumab before surgery.^13^

Despite a significant number of patients with durable response to immune checkpoint blockade, nearly half of all patients experience disease progression with either no response or with an initial response and development of resistance/progression. Currently, the etiology of resistance to immune checkpoint blockade is unknown, and there are no established biomarkers to predict response. Further understanding of the clinical features of patients with dMMR/MSI-H CRC who do not respond to immunotherapy is needed. In this study, we sought to evaluate patterns of disease progression (PD) in relation to survival among patients with advanced dMMR/MSI-H CRC treated with immunotherapy to better understand progression in this population and develop a framework to guide future studies and treatment selection based on these progression patterns.

## Methods

The Institutional Review Board of The University of Texas MD Anderson Cancer Center (UTMDACC) approved this retrospective analysis with an informed consent waiver. All patients from the MD Anderson Tumor Registry who presented with metastatic dMMR/MSI-H at the UTMDACC between January 2014 and December 2023 were identified. Patients were included in this study if they had documented metastatic disease, either at presentation or after initially presenting with localized disease, and received a PD-1 inhibitor, either alone or in combination with another immunotherapy agent.

Information regarding patient demographics, pathology, treatments administered, and treatment response was obtained from a review of the medical record. Designation of tumor stage was in accordance with the American Joint Cancer Committee on Cancer staging system and histologic grade was determined according to the World Health Organization standard grading system. Mismatch repair status was determined by immunohistochemical testing and MSI-H status was determined by PCR-based microsatellite instability testing or next-generation sequencing (NGS) performed in a CLIA-approved laboratory under routine clinical testing. Sporadic MSI-H was defined by the presence of somatic hyper-methylation of MLH-1 promoter or presence of a somatic *BRAF V600E* mutation whereas a hereditary MSI-H was defined as either the absence of MLH-1 promoter hypermethylation and *BRAF V600E* or the presence of a pathogenic germline mutations in MMR genes.

PD was defined as an increase in size of tumor(s) according to multi-disciplinary review of radiographic imaging (CT and/or MRI) which was obtained approximately every 8-12 weeks while patients were on treatment. PD was classified as either intrinsic, defined as progression at the first restaging scan, or adaptive, defined as progression after a best response of stable disease or responding disease. Patients with pseudoprogression (defined as radiographic PD on the first restaging scan but stable disease or better on the following restaging scan) were not included in the intrinsic group but, instead, were classified based on response after the initial psuedoprogression. Further, PD was classified as either single organ or systemic, defined as radiographic PD in more than one organ.

Time to progression (TTP) was defined as the time from initiation of immunotherapy to evidence of disease progression on serial radiographic imaging; patients were censored if no progression was seen on the last scan. Overall survival (OS) was defined as the time from initiation of immunotherapy until death of any cause; patients were censored if alive at last follow-up. Demographic and clinical variables were summarized using descriptive statistics, frequency (%) for categorical variables, and median (minimum, maximum) for continuous variables, grouped by tumor locations. Comparisons between continuous variables were conducted using the Mann–Whitney *U* test, while categorical groups were conducted using the nonparametric Fisher’s exact test when applicable. The Kaplan–Meier method was used to visualize and estimate survival curves. We applied a penalized Cox proportional hazard regression to identify the covariates that are most important to overall survival. (The following candidate variables were entered into the model - Age (tertiles); primary location (right/transverse vs left/rectal); differentiation (poor vs mod/well); primary intact (Y, N); stage at diagnosis (I-III vs IV); type of progression (intrinsic, adaptive); pattern of progression (single organ, systemic); site of progression (prior, new); RAS/BRAF mutation (RAS or BRAF V600e mutated vs neither/unknown); prior immunotherapy regimen prior to progression: nivo/ipi vs all others. We used the least absolute shrinkage and selection operator (LASSO) penalty and performed cross-validation to tune the penalty parameter. This procedure resulted in the selection of 3 covariates for the OS outcome: type of progression (intrinsic, adaptive); pattern of progression (single organ, systemic); RAS/BRAF mutation (RAS or BRAF V600e mutated vs neither/unknown). For TTP outcome, 2 covariates were selected: Differentiation (poor vs mod/well) and (intrinsic, adaptive). We then fit the unpenalized multivariate Cox model to estimate the hazard ratios of each feature. Our study was intended to be descriptive in nature as opposed to hypothesis-testing, and *P* value are presented without correction. For all analyses, a *P* value of ≤.05 was considered statistically significant. All statistical analyses were conducted using SPSS version 29.

## Results

We evaluated 166 metastatic MSI-H/dMMR colorectal cancer patients treated with immunotherapy from 2014 to 2023. At a median follow-up of 45.0 months, the median time to progression (mTTP) of all patients treated with immunotherapy was 86.0 months, and the median overall survival (mOS) of all patients treated with immunotherapy was not yet reached.

Table 1 presents the demographics and clinical features of all included patients. The median age was 59 years. The majority were male (55.4%) and Stage IV at initial presentation (53.6%). A majority of cases were determined to be MSI-H/dMMR by at least 2 methods (*n* = 95, 57%) with 27 (16%) being confirmed through 3 methods (IHC, PCR, NGS). Only 27 cases (16%) were determined using only one test and 2 (1%) were determined through genetic testing for germline mutation. The most common sites of metastatic disease involvement were distant lymph nodes (65.1%), peritoneum (36.7%), and liver (35.5%). The majority of patients did not have the primary tumor intact at the time of the analysis (74.1%). Most patients had not received prior chemotherapy (66.3%). The most common immunotherapy regimens administered were single-agent pembrolizumab (50%), single-agent nivolumab (16.9%), and nivolumab plus ipilimumab (16.9%).

**Table 1.** Patient demographics and clinical features at the initiation of immune checkpoint inhibitor (ICB).

Patient demographics (*n* = 166)		
**Age**	Median	59
	Range	19-97
**Sex**	Male	92 (55.4%)
	Female	74 (44.6%)
**Race**	Asian	6 (3.6%)
	Black or African American	13 (7.8%)
	White	129 (77.7%)
	Other	15 (9.0%)
	Unknown	3 (1.8%)

Clinical features (*n* = 166)		
**Stage at diagnosis**	I, II, III	76 (45.8%)
	IV	89 (53.6%)
	Unknown	1 (0.6%)
**Differentiation**	Poor	86 (51.8%)
	Moderate	74 (44.6%)
	Well	0 (0%)
	Unknown	6 (3.6%)
**Location of primary**	Ascending colon	87 (52.4%)
	Transverse colon	22 (13.3%)
	Descending colon	37 (22.3%)
	Rectum	17 (10.2%)
	>1 location	3 (1.8%)
**Primary tumor intact at initiation of ICB**	Yes	43 (25.9%)
	No	123 (74.1%)
**Site of metastatic disease at initiation of ICB**	Lung	24 (14.5%)
	Liver	59 (35.5%)
	Distant lymph node	108 (65.1%)
	Peritoneum	61 (36.7%)
	Other	51 (30.7%)
**Etiology of dMMR/MSI-H status**	Germline	46 (27.7%)
	Sporadic	74 (44.6%)
	Unknown	46 (27.7%)
**Tumor mutational burden**	Low (<10)	14 (8.4%)
	High (≥10)	119 (71.7%)
	Unknown	33 (19.9%)
***RAS* mutation status**	NRAS mutation	4 (2.4%)
	KRAS mutation	62 (37.3%)
	Wild type	75 (45.1%)
	Unknown	23 (13.9%)
***BRAF V600E* mutation status**	Mutated	41 (24.7%)
	Wild type	107 (64.5%)
	Unknown	18 (10.8%)

Treatment (*n* = 166)		
**Prior chemotherapy**	Yes	56 (33.7%)
	No	110 (66.3%)
**Immunotherapy agent**	Nivolumab	28 (16.9%)
	Pembrolizumab	83 (50.0%)
	Durvalumab	2 (1.2%)
	Atezolizumab	7 (4.2%)
	Nivolumab + ipilimumab	28 (16.9%)
	Single-agent ICB + vaccine	5 (3.0%)
	Single-agent ICB + chemotherapy	4 (2.4%)
	Other ICB	9 (5.4%)
**Progression on immunotherapy**	Yes	64 (38.6%)
	No	102 (61.4%)

**Figure 1.**
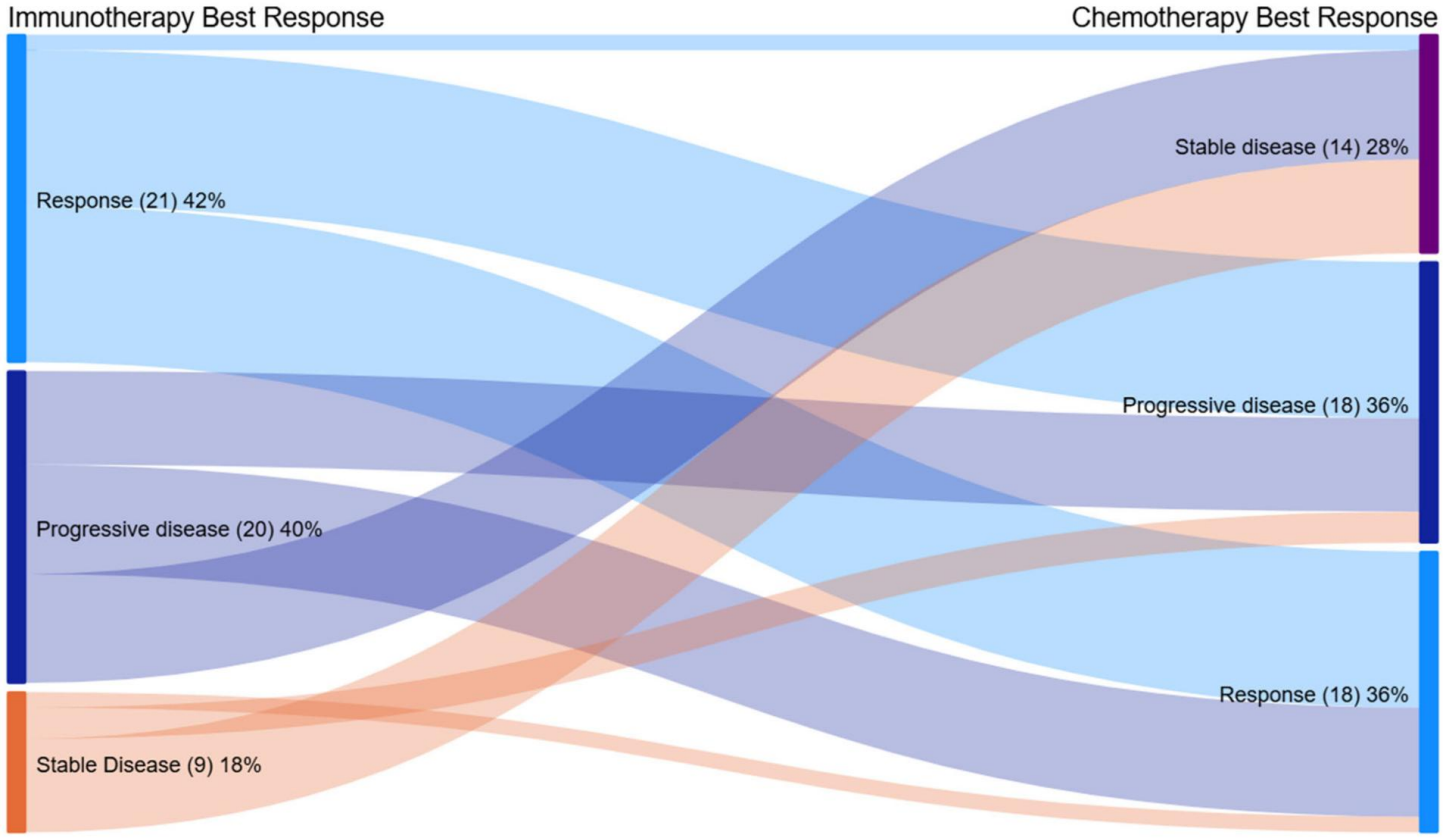
Sankey diagram of ICB response and response to chemotherapy.

Progression on immunotherapy was seen in 64 patients (38.6%). To understand the relationship between immunotherapy and chemotherapy response, we analyzed the best response status for the first chemotherapy and immunotherapy given to patients for metastatic disease (Figure 1). A total of 56 patients received both for metastatic disease with 10 receiving immunotherapy first and 46 receiving chemotherapy first. Progressive disease for either immunotherapy or chemotherapy did not predict for progression for the other therapy, respectively. Of the 20 patients with best response of progression to immunotherapy 7 (35%) demonstrated responsive disease to chemotherapy. For the 18 patients with best response of progression to chemotherapy, 10 (55%) demonstrated response to immunotherapy.

Median TTP for all patients was 86.4 months (95% CI 25.23-147.5 months). The characteristics and details of the 64 patients with PD are displayed in Table 2. Among patients who had PD, 87.5% had PD exclusively at metastatic sites, while 6.3% had PD at the primary site alone, with the majority of progression occurring at sites of prior disease (76.6%). A similar distribution between single organ progression (48.4%) and systemic progression (51.6%) was observed. Post-progression therapy included systemic therapy in 56.3% and local modality therapy in 28.2%. Local modality therapy was utilized in 42% of single organ progression cases compared to 15% of systemic progression cases. There was a similar rate of adaptive PD (50.0%) and intrinsic PD (50.0%). A systemic progression pattern was more commonly seen in intrinsic PD (62.5%), than in adaptive PD (40.6%). In adaptive progression cases, those with local modality treatment had a trend toward improved OS compared to those without local treatment, but it was not statistically significant (60.2 months vs 39.5 months, *P* = .853).

**Table 2.** Clinical features of patients with radiographic PD on ICB.

Clinical features of patients who had PD on immunotherapy (*n* = 64)		
**Site of PD**	Primary	4 (6.3%)
	Metastatic	56 (87.5%)
	Both	4 (6.3%)
**Location of PD**	New site	4 (6.8%)
	Prior site	49 (76.6%)
	Both	11 (17.2%)
**Extent of progression**	Single organ progression	31 (48.4%)
	Systemic progression	33 (51.6%)
**Mechanism of progression**	Adaptive	32 (50.0%)
	Intrinsic	32 (50.0%)
***BRAF V600E* status**	Mutated	15 (23.4%)
	Wild type	45 (70.3%)
	Unknown	4 (6.3%)
***KRAS* status**	Mutated	24 (37.5%)
	Wild type	40 (62.5%)

Treatment (*n* = 66)		
**Treatment after progression**	Surgery	6 (9.4%)
	Other local therapy	12 (18.8%)
	Systemic therapy	36 (56.3%)
	Lost to follow-up or death	10 (15.6%)
**Type of systemic therapy after progression (*n* = 36)**	Dual ICB	8 (22.2%)
	Single agent ICB	6 (9.1%)
	ICB + other combo	11 (16.7%)
	Chemotherapy	11 (30.6%)

**Figure 2.**
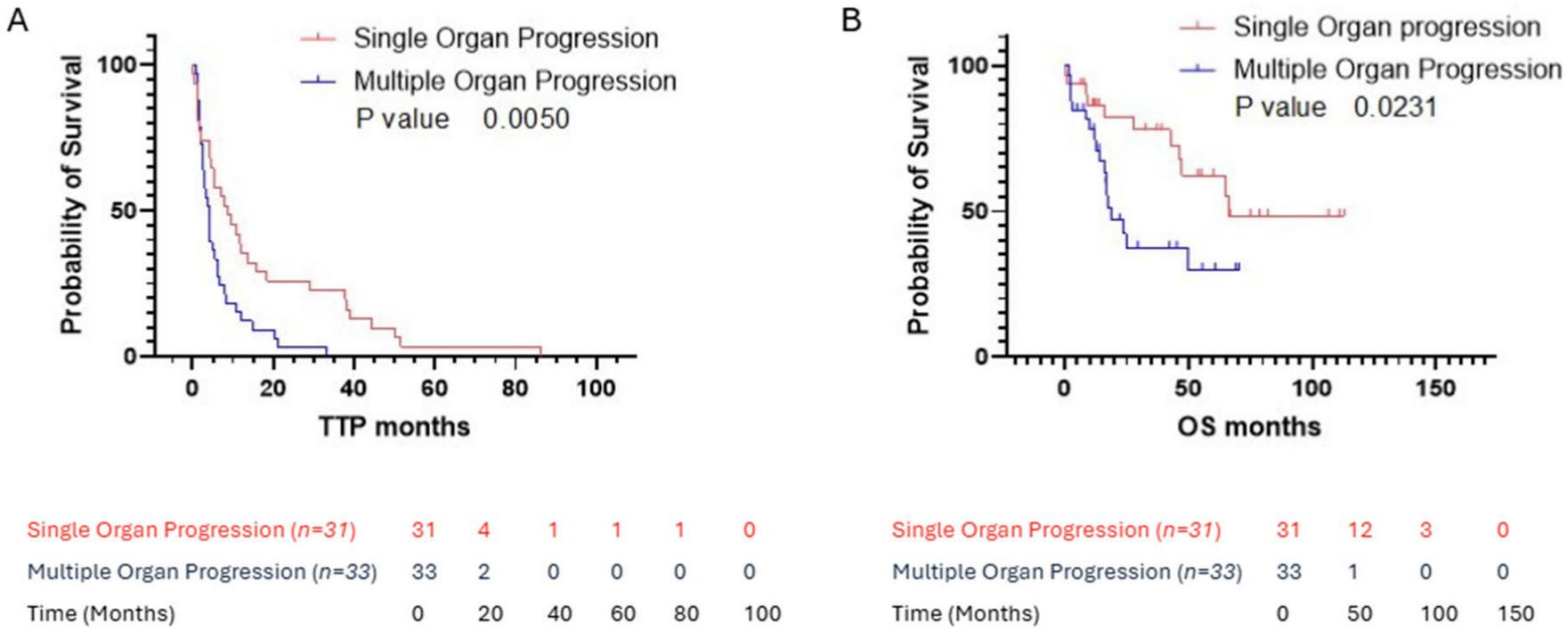
Outcomes in patients with single organ progression vs multiple organ progression. (A) TTP. (B) OS.

Figure 2A and B shows TTP and OS, respectively, in patients who had PD based on whether they had single organ vs systemic PD. Median TTP for 31 patients with single organ PD was 8.8 months vs 4.0 months for 33 patients with systemic PD (*P* = .005; Figure 2A). Median OS was 65.9 months for 31 patients with single organ PD vs 18.7 months for 33 patients with systemic PD (*P* = .023; Figure 2B).

**Figure 3.**
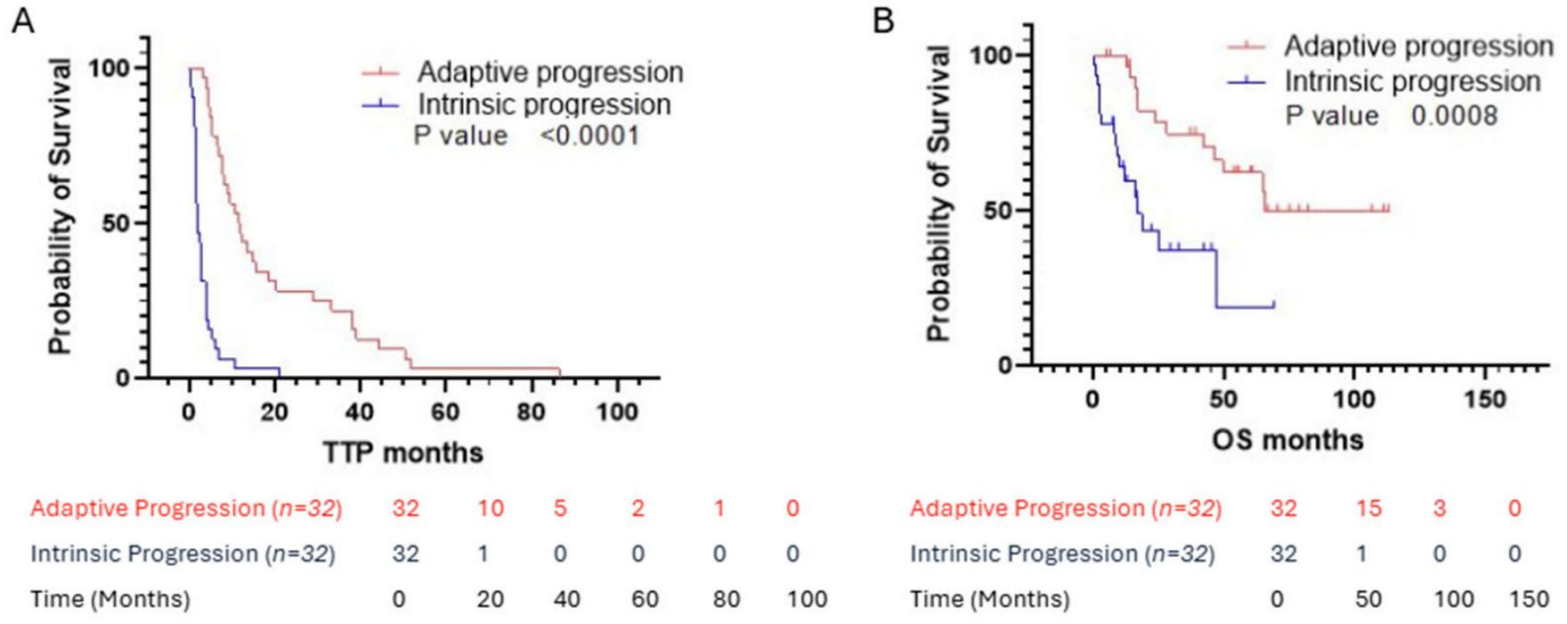
Outcomes in patients with adaptive progression vs intrinsic progression. (A) TTP. (B) OS.

Figure 3A and B shows TTP and OS, respectively, based on whether patients had adaptive or intrinsic PD. Median TTP was 11.6 months for patients with adaptive PD vs 1.9 months for patients with intrinsic PD (*P* = <.01; Figure 3A). Median OS was 65.9 months for patients with adaptive PD vs 17.0 months for intrinsic PD (*P* = .001; Figure 3B).

**Figure 4.**
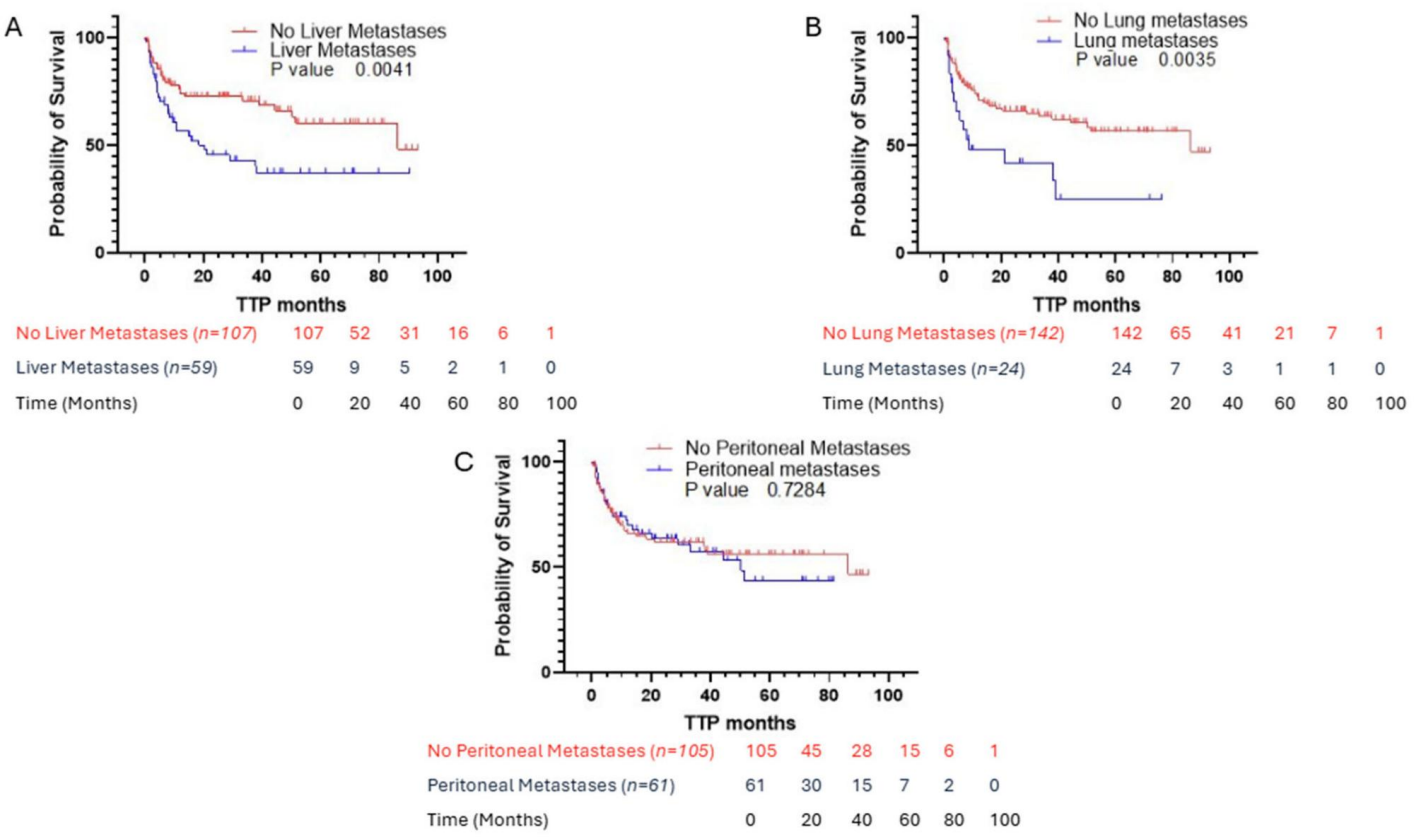
TTP by site of metastatic disease. (A) Patients with liver metastases vs without liver metastases. (B) Patients with lung metastases vs without lung metastases. (C) Patients with peritoneal disease vs without peritoneal disease.

Figure 4A-C shows TTP based on the presence of liver, lung (with lymph node only), and peritoneal metastases, respectively. Median TTP was 20.4 months for 59 patients with liver metastases vs 86.4 months for 107 patients with non-liver metastases (*P* = .004; Figure 4A). Median TTP was 8.8 months for 24 patients with lung metastases vs 86.4 months for 142 patients with non-lung metastases (*P* = .003; Figure 4B). Median TTP was 50.3 months for 61 patients with peritoneal metastases, with 25 events, vs 86.4 months for 105 patients with non-peritoneal metastases (*P* = .728; Figure 4C).

A multivariate Cox proportional hazards regression analysis was conducted to identify independent predictors of overall survival and progression. Intrinsic progression was significantly associated with worse overall survival, with a HR of 7.23 (95% CI: 2.34-22.28; *P *= .001), indicating a strong and statistically significant prognostic effect. Systemic progression was also associated with a poorer overall survival in univariate analysis but was not statistically significant in multivariate analysis (HR: 1.54, 95% CI: 0.61-3.92; *P *= .363). Of note, patients with BRAF wild type (WT) status has statistically significantly improved TTP compared to those with BRAF V600E mutations (Supplemenary Figure 1) while no statistical difference was seen in KRAS WT vs KRAS mutated (Supplementary Figure 2). There was also no stasitical difference in TPP or OS based on immunotherapy regimen (Supplementary Figures 3, 4 resepectively).

## Discussion

Our retrospective analysis of 166 patients with metastatic dMMR/MSI-H CRC treated at MD Anderson Cancer Center from 2014 to 2023 identified patterns of PD and survival in patients with dMMR/MSI-H CRC treated with immunotherapy. This study introduces a nuanced portrait and a novel framework that can be used to guide future prospective studies into patterns of PD and the natural history of disease among patients with this subtype of CRC.

At a median follow-up of 45.0 months, approximately 39% of patients in our study had any type of PD, which is similar to rates of PD among patients enrolled in KEYNOTE 177 (2-year PFS rate of 48% and 5-year PFS rate of 34%) and Checkmate 142 (60-month PFS rate of 55%).^8^^,^^14^ The clinicodemographic features of patients in our analysis, particularly age, sex, location of the primary tumor, and mutational status, are similar to those in KEYNOTE 177 and Checkmate 142.^8–10^

The majority of the 64 patients who had PD in our analysis did so in metastatic sites (87.5%) rather than in the primary tumor. Several hypotheses surrounding the mechanisms underlying PD have been posited.^14^ However, a clear model of progression has not been established for dMMR/MSI-H CRC. Single organ and systemic PD occurred in approximately the same number of unique patients (31 and 33, respectively). Patients with single organ PD had a notably longer OS compared to those with systemic progression, which could have been driven by the more frequent utilization of local modality therapy or more indolent cancer behavior. This should reinforce the clinical consideration for local modality therapy in these patients whether that be surgery, radiation, or interventional radiology interventions.

Intrinsic and adaptive PD occurred in approximately the same number of unique patients (32 vs 32, respectively). Unsurprisingly, patients with intrinsic PD had shorter TTP than patients with adaptive PD and shorter OS. Mechanisms of intrinsic resistance to immunotherapy in patients with dMMR/MSI-H CRC are thought to be multifactorial and relating to ability to illicit an immune response.^14^ On the other hand, adaptive resistance may be related to different mechanisms. Due to the poor outcomes of intrinsic progressors, there should be a low threshold to engage new treatments through clinical trials such as Werner Syndrome helicase (WRN) inhibitors which have shown to have activity in MSI-H colorectal cancer with resistance to ICB.^15^ Further study is needed to understand the mechanisms of resistance and to develop these innovative treatments for this population of intrinsic progressors.

Patients who had liver metastases had shorter mTTP and mOS compared to those without liver metastases. Oher studies have shown patients with metastatic dMMR/MSI-H CRC treated with immunotherapy, the presence of liver metastases, but not other sites (including non-regional lymph node, lung, and peritoneum), was associated with shorter PFS and an attenuated best tumor response (HR 2.82; 95% CI 1.08-7.39; *P* = .03).^16^ Patients with liver metastasis had a mPFS of 6 months compared to 34 months among patients without liver metastasis.^16^

Further, among patients with PD, those harboring a *BRAF V600E* mutation had shorter mTTP. This is not a replicated finding of either Keynote 177 or Checkmate 142 where we see no statistically significant difference in clinical outcomes between patients with BRAF V600E mutated disease and those without.^8–10^ The number of patients with TMB of 10 mut/mB or greater was low in our analysis, mostly related to incomplete data capture of this variable. However, in a study of 22 patients with metastatic dMMR/MSI-H CRC treated with immunotherapy, TMB showed a strong association with objective response (*P* < .001) and PFS (*P* < .001).^17^

The limitations of our study include its retrospective nature and restriction to patients treated at one institution, which may introduce biases and limit generalizability. Additionally, the lack of granularity pertaining to some clinicodemographic and tumoral features led to a relatively high percentage of “unknowns” (eg, for TMB), which constrains the comprehensive interpretation of our data. Further, PD was determined using multidisciplinary review, and pseudoprogression was a subjective determination. In a report of 123 patients with metastatic dMMR/MSI-H CRC treated with immunotherapy, 12 patients (9.8%) had pseudoprogression in the first 3 months of treatment.^18^ To mitigate the possibility of categorizing patients who experienced pseudoprogression into the intrinsic PD category, patients who had PD on the first restaging scan but stable disease or better on the following restaging scan after being continued on immunotherapy were either considered responders or adaptive progressors. It is possible that utilizing iRECIST during the first 3 months could have led to a slightly different proportion of patients who were deemed to have intrinsic and adaptive PD. We also acknowledge that when evaluating progression at the primary and metastatic sites, there is a limitation to radiographic evaluation of primaries and endoscopic evaluation was not available for the majority of patients.

## Conclusions

Our study highlights the various patterns of PD and their impact on survival in patients with dMMR/MSI-H CRC. Our findings suggest tailoring therapeutic approaches, particularly for patients with single organ disease. Local therapies may increase survival in patients with single organ or oligometastatic progression. Considering that patients with intrinsic and systemic progression have worse outcomes, these progression patterns identify a patient population who may benefit through escalated/alterative systemic therapies and innovative treatments through clinical trials. Moving forward, prospective studies with longitudinal monitoring of the immune profile could provide insights into the temporal dynamics of the tumor-immune interaction that presumably drives resistance to immunotherapy in patients with dMMR/MSI-H CRC. Understanding the mechanisms behind intrinsic PD is urgent as immunotherapy becomes the standard treatment paradigm in the neoadjuvant setting for patients with localized and locally advanced dMMR/MSI-H CRC.

## Supplementary Material

oyag235_Supplementary_Data

## Data Availability

The datasets generated and/or analyzed during the current study are available from the corresponding author upon reasonable request.
