# Supplementary material for: Patterns of progression and survival in patients with mismatch repair deficient/ microsatellite instability- high metastatic colorectal cancer treated with immunotherapy
Source: Oncologist. 2026 Jul 20;31(8):oyag235. doi: 10.1093/oncolo/oyag235 (PMC13399441; doi:10.1093/oncolo/oyag235)

Supplemental Figure 1A – TTP in patients who had PD based on *BRAFV600E* status


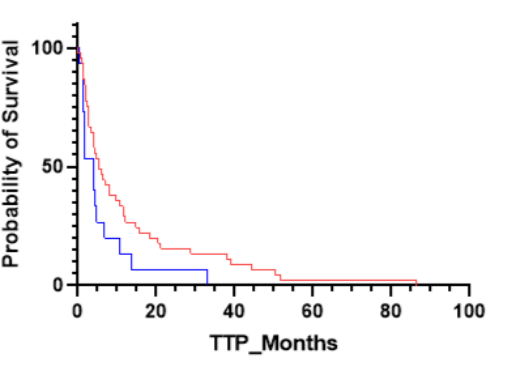


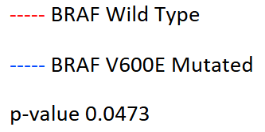


Supplemental Figure 2A – TTP in patients who had PD based on *RAS* status
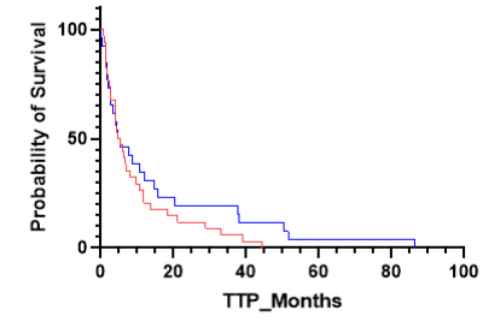


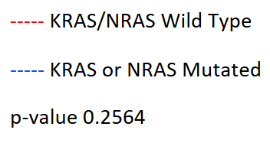


Supplemental Figure 3A- TTP by immunotherapy Regimen


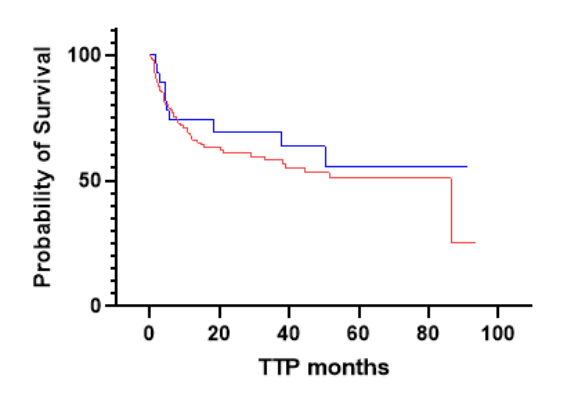


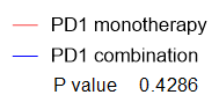


Supplemental Figure 3B- OS by immunotherapy Regimen


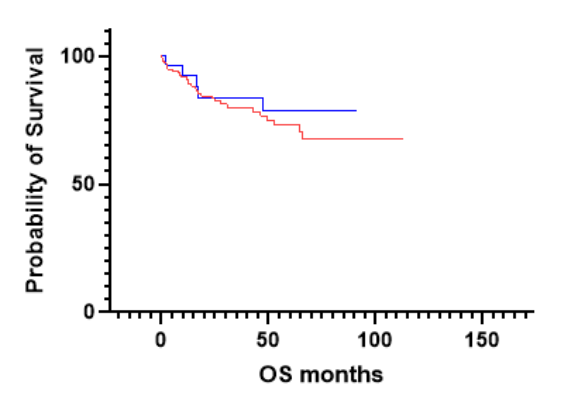


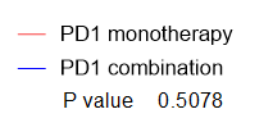

Supplement: oyag235_Supplementary_Data [file oyag235_supplementary_data.docx]
